# Supplementary material for: LP.8.1-directed COVID-19 mRNA vaccines durably boost neutralizing antibodies and mitigate ancestral immune imprinting
Source: PLoS Pathog. 2026 May 11;22(5):e1014218. doi: 10.1371/journal.ppat.1014218 (PMC13178986; doi:10.1371/journal.ppat.1014218)
Supplement: S1 Table — No., number; y.o., years old; WT, wildtype; MV, monovalent vaccine; BV, bivalent vaccine. (DOCX) [file ppat.1014218.s001.docx]

|  | | **All participants** | | **Age Cohort 1 (18-49 y.o.)** | | **Age Cohort 2 (50-64 y.o.)** | | **Age Cohort 3 (65+ y.o.)** | |  |
| --- | --- | --- | --- | --- | --- | --- | --- | --- | --- | --- |
|  |  | No. or Mean | % or (range) | No. or Mean | % or (range) | No. or Mean | % or (range) | No. or Mean | % or (range) |  |
|  |  |  |  |  |  |  |  |  |  |  |
| **Total** | | 36 | - | 18 | - | 10 | - | 8 | - |  |
| **Female** | | 28 | 77.8% | 12 | 66.7% | 9 | 90% | 7 | 87.5% |  |
| **Male** | | 7 | 19.4% | 5 | 27.8% | 1 | 10% | 1 | 12.5% |  |
| **Prefer Not to Answer** | | 1 | 2.8% | 1 | 5.56% | - | - | - | - |  |
| **Age** | | 47.3 | (19, 80) | 30.4 | (19, 48) | 58.1 | (52, 63) | 71.9 | (66, 80) |  |
| **No. Vaccines** | All vaccines | 6.7 | (4, 10) | 5.9 | (5, 7) | 7 | (4, 8) | 8 | (4, 10) |  |
|  | WT | 3.3 | (2, 4) | 3 | (3, 3) | 3.4 | (2, 4) | 3.6 | (2, 4) |  |
|  | BA.5 BV | 0.8 | (0, 2) | 0.6 | (0, 1) | 1 | (0, 1) | 0.9 | (0, 2) |  |
|  | XBB.1.5 | 0.7 | (0, 2) | 0.4 | (0, 1) | 0.8 | (0, 1) | 1.1 | (0, 2) |  |
|  | KP. 2 MV | 0.9 | (0,2) | 0.8 | (0, 1) | 0.8 | (0, 1) | 1.4 | (1, 2) |  |
|  | LP.8.1 MV | 1.0 | (1,1) | 1 | (1, 1) | 1 | (1, 1) | 1 | (1, 1) |  |
| **Sera Days Post Infection (Pre)** | | 796.0 | (0, 1626) | 874.6 | (0, 833.7) | 829.7 | (0, 1235) | 668.2 | (0, 1049) |  |
| **Sera Days Post Infection (Post)** | | 831.6 | (0, 1674) | 871.9 | (0, 1391) | 865.5 | (0, 1276) | 697 | (0, 1077) |  |
| **Sera Days Pre LP.8.1 MV Vaccination** | | 6.1 | (0, 28) | 6.6 | (0, 28) | 6.8 | (1, 16) | 4.1 | (0, 25) |  |
| **Sera Days Post LP.8.1 MV Vaccination** | | 28.6 | (21, 45) | 30.6 | (21, 45) | 26.3 | (22, 34) | 27 | (23, 33) |  |
