# Supplementary material for: LP.8.1-directed COVID-19 mRNA vaccines durably boost neutralizing antibodies and mitigate ancestral immune imprinting
Source: PLoS Pathog. 2026 May 11;22(5):e1014218. doi: 10.1371/journal.ppat.1014218 (PMC13178986; doi:10.1371/journal.ppat.1014218)
Supplement: S2 Table — Vaccine formulations are denoted as wildtype (WT), BA.5 Bivalent (BA.5), XBB.1.5 monovalent (XBB.1.5), and KP.2 monovalent (KP.2). Vaccine manufacturers are denoted as Pfizer (P), Moderna (M), and Unknown (U). Yr, years; Infx, infection; Vax, vaccination; DBV, days before vaccination; DPV, days post vaccination; DPI, days post infection; F, female; M, male; Wh, white; Af, Black or African American; As, Asian. (DOCX) [file ppat.1014218.s002.docx]

| **ID** | **Age (Yr)** | **Sex** | **Race** | **No. Vax** | **No. WT Vax** | **No. BA.5 Bivalent Vax** | **No XBB.1.5 Vax** | **No. KP.2 MV** | **No. LP.8.1 MV** | **Sera Days Post Most Recent Infx  Pre** | **Sera Days Post Most Recent Infx  Post** | **Sera Days  Pre LP.8.1 MV** | **Sera Days  Post LP.8.1 MV** | **Vaccine History** |
| --- | --- | --- | --- | --- | --- | --- | --- | --- | --- | --- | --- | --- | --- | --- |
| CUMC 1 | 26 | F | Asian | 7 | 3 | 1 | 1 | 1 | 1 | NA | NA | 2 | 28 | WT-P/WT-P/WT-P/BA.5-M/XBB.1.5-M/KP.2-M/LP.8.1-M |
| CUMC 3 | 22 | M | Asian | 5 | 3 | 0 | 0 | 1 | 1 | 141 | 175 | 3 | 31 | WT-P/WT-P/WT-P/KP.2-P/LP.8.1-P |
| CUMC 8 | 23 | F | Asian | 5 | 3 | 0 | 0 | 1 | 1 | NA | NA | 1 | 31 | WT-P/WT-P/WT-P/KP.2-M/LP.8.1-M |
| CUMC 16 | 23 | M | Asian | 4 | 3 | 0 | 0 | 0 | 1 | 1030 | 1063 | 1 | 32 | WT-P/WT-P/WT-P/LP.8.1-P |
| CUMC 18 | 23 | F | Asian | 4 | 3 | 0 | 0 | 0 | 1 | NA | NA | 2 | 31 | WT-S/WT-S/WT-S/LP.8.1-P |
| MICH 1 | 48 | F | White | 5 | 3 | 0 | 0 | 0 | 1 | 896 | 927 | 0 | 31 | WT-P/WT-P/WT-P/XBB.1.5-P/LP.8.1-P |
| MICH 2 | 30 | F | Black or African American | 6 | 3 | 1 | 0 | 1 | 1 | 1107 | 1148 | 7 | 34 | WT-P/WT-P/WT-P/BA.5-P/KP.2-P/LP.8.1-P |
| MICH 3 | 31 | M | White | 6 | 3 | 0 | 1 | 1 | 1 | 901 | 953 | 28 | 24 | WT-P/WT-P/WT-P/XBB.1.5-P/KP.2-P/LP.8.1-M |
| MICH 4 | 75 | F | More than One Race | 10 | 4 | 2 | 1 | 2 | 1 | NA | NA | 1 | 26 | WT-M/WT-M/WT-M/WT-M/BA.5-M/BA.5-M/XBB.1.5-M/KP.2-N/KP.2-P/LP.8.1-M |
| MICH 5 | 33 | F | White | 7 | 3 | 1 | 1 | 1 | 1 | 260 | 286 | 1 | 25 | WT-P/WT-P/WT-P/BA.5-P/XBB.1.5-P/KP.2-M/LP.8.1-M |
| MICH 6 | 26 | F | White | 5 | 3 | 1 | 0 | 0 | 1 | 1072 | 1115 | 9 | 34 | WT-P/WT-P/WT-P/BA.5-P/LP.8.1-M |
| MICH 7 | 52 | F | White | 7 | 3 | 1 | 1 | 1 | 1 | NA | NA | 10 | 25 | WT-P/WT-P/WT-P/BA.5-P/XBB.1.5-M/KP.2-M/LP.8.1-M |
| MICH 8 | 55 | F | White | 8 | 4 | 1 | 1 | 1 | 1 | 742 | 778 | 4 | 32 | WT-P/WT-P/WT-P/WT-P/BA.5-P/XBB.1.5-P/KP.2-N/LP.8.1-M |
| MICH 9 | 29 | F | White | 6 | 3 | 1 | 0 | 1 | 1 | 430 | 473 | 9 | 34 | WT-P/WT-P/WT-P/BA.5-U/KP.2-U/LP.8.1-P |
| MICH 10 | 60 | F | White | 7 | 3 | 1 | 1 | 1 | 1 | 1235 | 1276 | 7 | 34 | WT-M/WT-M/WT-M/BA.5-M/XBB.1.5-M/KP.2-M/LP.8.1-M |
| MICH 11 | 60 | F | White | 6 | 3 | 1 | 0 | 1 | 1 | 1177 | 1220 | 16 | 27 | WT-M/WT-M/WT-M/BA.5-P/KP.2-M/LP.8.1-M |
| MICH 12 | 29 | F | White | 7 | 3 | 1 | 1 | 1 | 1 | 925 | 964 | 8 | 31 | WT-P/WT-P/WT-P/BA.5-P/XBB.1.5-P/KP.2-P/LP.8.1-P |
| MICH 13 | 38 | F | White | 7 | 3 | 1 | 1 | 1 | 1 | 824 | 856 | 0 | 32 | WT-P/WT-P/WT-P/BA.5-P/XBB.1.5-P/KP.2-P/LP.8.1-M |
| MICH 14 | 63 | F | White | 4 | 2 | 1 | 0 | 0 | 1 | NA | NA | 1 | 22 | WT-P/Wt-M/BA.5-M/LP.8.1-P |
| MICH 15 | 46 | F | White | 6 | 3 | 1 | 0 | 1 | 1 | 1626 | 1674 | 3 | 45 | WT-P/WT-P/WT-P/BA.5-P/KP.2-P/LP.8.1-P |
| MICH 16 | 37 | F | White | 7 | 3 | 1 | 1 | 1 | 1 | 190 | 239 | 28 | 21 | WT-P/WT-P/WT-P/BA.5-P/XBB.1.5-P/KP.2-P/LP.8.1-P |
| MICH 17 | 59 | M | White | 8 | 4 | 1 | 1 | 1 | 1 | NA | NA | 3 | 24 | WT-M/WT-M/WT-M/WT-M/BA.5-M/XBB.1.5-M/KP.2-M/LP.8.1-M |
| MICH 18 | 72 | M | White | 10 | 4 | 1 | 2 | 2 | 1 | 479 | 502 | 0 | 23 | WT-M/WT-M/WT-M/WT-M/BA.5-M/XBB.1.5-M/XBB.1.5-N/KP.2-M/KP.2-M/LP.8.1-M |
| MICH 19 | 33 | O | White | 7 | 3 | 1 | 1 | 1 | 1 | 748 | 784 | 2 | 34 | WT-M/Wt-M/WT-M/BA.5-U/XBB.1.5-U/KP.2-U/LP.8.1-M |
| MICH 20 | 57 | F | White | 6 | 3 | 1 | 1 | 0 | 1 | 1110 | 1138 | 1 | 27 | WT-P/WT-P/WT-M/BA.5-M/XBB.1.5-P/LP.8.1-M |
| MICH 21 | 31 | M | White | 5 | 3 | 0 | 0 | 1 | 1 | 1355 | 1391 | 13 | 23 | WT-P/WT-P/WT-M/KP.2-M/LP.8.1-P |
| MICH 22 | 19 | M | More than One Race | 7 | 3 | 1 | 1 | 1 | 1 | 1000 | 1031 | 1 | 30 | WT-P/WT-P/WT-P/BA.5-P/XBB.1.5-P/KP.2-M/LP.8.1-P |
| MICH 23 | 73 | F | White | 8 | 4 | 1 | 1 | 1 | 1 | 197 | 228 | 1 | 30 | WT-P/WT-P/WT-P/WT-P/BA.5-P/XBB.1.5-M/KP.2-M/LP.8.1-M |
| MICH 24 | 66 | F | White | 10 | 4 | 1 | 2 | 2 | 1 | 576 | 609 | 0 | 33 | WT-P/WT-P/WT-P/WT-P/BA.5-P/XBB.1.5-P/XBB.1.5-P/KP.2-M/KP.2-P/LP.8.1-M |
| MICH 25 | 60 | F | White | 8 | 4 | 1 | 1 | 1 | 1 | 480 | 510 | 7 | 23 | WT-M/WT-M/WT-M/WT-M/BA.5-M/XBB.1.5-P/KP.2-M/LP.8.1-P |
| MICH 26 | 73 | F | White | 7 | 3 | 1 | 1 | 1 | 1 | N/A | N/A | 25 | 24 | WT-P/WT-P/WT-P/BA.5-P/XBB.1.5-P/KP.2-P/LP.8.1-P |
| MICH 27 | 80 | F | White | 8 | 4 | 1 | 1 | 1 | 1 | 1049 | 1077 | 1 | 27 | WT-P/WT-P/WT-P/WT-P/BA.5-M/XBB.1.5-M/KP.2-M/LP.8.1-M |
| MICH 28 | 56 | F | Asian | 8 | 4 | 1 | 1 | 1 | 1 | NA | NA | 4 | 27 | WT-M/WT-M/WT-M/WT-M/BA.5-P/XBB.1.5-M/KP.2-N/LP.8.1-M |
| MICH 29 | 68 | F | White | 7 | 4 | 0 | 1 | 1 | 1 | 666 | 696 | 0 | 30 | WT-P/WT-P/WT-M/WT-M/XBB.1.5-M/KP.2-M/LP.8.1-M |
| MICH 30 | 68 | F | White | 4 | 2 | 0 | 0 | 1 | 1 | 1042 | 1070 | 5 | 23 | WT-J/WT-M/KP.2-M/LP.8.1-M |
| MICH 31 | 59 | F | White | 8 | 4 | 1 | 1 | 1 | 1 | 234 | 271 | 15 | 22 | WT-M/WT-M/WT-M/WT-M/BA.5-M/XBB.1.5-P/KP.2-P/LP.8.1-M |
