# Supplementary material for: LP.8.1-directed COVID-19 mRNA vaccines durably boost neutralizing antibodies and mitigate ancestral immune imprinting
Source: PLoS Pathog. 2026 May 11;22(5):e1014218. doi: 10.1371/journal.ppat.1014218 (PMC13178986; doi:10.1371/journal.ppat.1014218)
Supplement: S3 Table — No., number; y.o., years old; WT, wildtype; MV, monovalent vaccine; BV, bivalent vaccine; 1m, ~ 1 month post-boost sample; 4m, ~ 4 month post-boost sample. (DOCX) [file ppat.1014218.s003.docx]

|  | | **All participants** | |  |
| --- | --- | --- | --- | --- |
|  |  | No. or Mean | % or (range) |  |
|  |  |  |  |  |
| **Total** | | 11 | - |  |
| **Female** | | 6 | 54.5% |  |
| **Male** | | 4 | 36.4% |  |
| **Prefer Not to Answer** | | 1 | 9.1% |  |
| **Age** | | 36.0 | (22, 72) |  |
| **No. Vaccines** | All vaccines | 6.3 | (4, 10) |  |
|  | WT | 3.2 | (2, 4) |  |
|  | BA.5 BV | 0.6 | (0, 2) |  |
|  | XBB.1.5 | 0.6 | (0, 2) |  |
|  | KP. 2 MV | 0.8 | (0,2) |  |
|  | LP.8.1 MV | 1.0 | (1,1) |  |
| **Sera Days Post Infection (1m)** | | 794.3 | (0, 1291) |  |
| **Sera Days Post Infection (4m)** | | 869.9 | (0, 1354) |  |
| **Sera Days Pre LP.8.1 MV Vaccination (1m)** | | 29.5 | (24, 36) |  |
| **Sera Days Post LP.8.1 MV Vaccination (4m)** | | 111.7 | (85, 156) |  |
