# Supplementary material for: LP.8.1-directed COVID-19 mRNA vaccines durably boost neutralizing antibodies and mitigate ancestral immune imprinting
Source: PLoS Pathog. 2026 May 11;22(5):e1014218. doi: 10.1371/journal.ppat.1014218 (PMC13178986; doi:10.1371/journal.ppat.1014218)
Supplement: S4 Table — Vaccine formulations are denoted as wildtype (WT), BA.5 Bivalent (BA.5), XBB.1.5 monovalent (XBB.1.5), and KP.2 monovalent (KP.2). Vaccine manufacturers are denoted as Pfizer (P), Moderna (M), and Unknown (U). Yr, years; Infx, infection; Vax, vaccination; DBV, days before vaccination; DPV, days post vaccination; DPI, days post infection; F, female; M, male; Wh, white; Af, Black or African American; As, Asian. (DOCX) [file ppat.1014218.s004.docx]

| **ID** | **Age (Yr)** | **Sex** | **Race** | **No. Vax** | **No. WT Vax** | **No. BA.5 Bivalent Vax** | **No XBB.1.5 Vax** | **No. KP.2 MV** | **No. LP.8.1 MV** | **Sera Days Post Most Recent Infx  (1m)** | **Sera Days Post Most Recent Infx  (4m)** | **Sera Days  Post LP.8.1 MV (1m)** | **Sera Days Post LP.8.1 MV (4m)** | **Vaccine History** |
| --- | --- | --- | --- | --- | --- | --- | --- | --- | --- | --- | --- | --- | --- | --- |
| CUMC 1 | 26 | F | Asian | 7 | 3 | 1 | 1 | 1 | 1 | NA | NA | 28 | 152 | WT-P/WT-P/WT-P/BA.5-M/XBB.1.5-M/KP.2-M/LP.8.1-M |
| CUMC 3 | 22 | M | Asian | 5 | 3 | 0 | 0 | 1 | 1 | 175 | 300 | 31 | 156 | WT-P/WT-P/WT-P/KP.2-P/LP.8.1-P |
| CUMC 8 | 23 | F | Asian | 5 | 3 | 0 | 0 | 1 | 1 | NA | NA | 31 | 142 | WT-P/WT-P/WT-P/KP.2-M/LP.8.1-M |
| CUMC 16 | 23 | M | Asian | 4 | 3 | 0 | 0 | 0 | 1 | 1063 | 1173 | 32 | 142 | WT-P/WT-P/WT-P/LP.8.1-P |
| CUMC 17 | 22 | F | Asian | 5 | 3 | 0 | 0 | 1 | 1 | 1291 | 1354 | 36 | 99 | WT-P/WT-P/WT-P/KP.2-P |
| MICH 5 | 33 | F | White | 7 | 3 | 1 | 1 | 1 | 1 | 286 | 357 | 25 | 96 | WT-P/WT-P/WT-P/BA.5-P/XBB.1.5-P/KP.2-M/LP.8.1-M |
| MICH 6 | 26 | F | White | 5 | 3 | 1 | 0 | 0 | 1 | 1115 | 1171 | 34 | 90 | WT-P/WT-P/WT-P/BA.5-P/LP.8.1-M |
| MICH 17 | 59 | M | White | 8 | 4 | 1 | 1 | 1 | 1 | NA | NA | 24 | 88 | WT-M/WT-M/WT-M/WT-M/BA.5-M/XBB.1.5-M/KP.2-M/LP.8.1-M |
| MICH 18 | 72 | M | White | 10 | 4 | 1 | 2 | 2 | 1 | 502 | 568 | 23 | 89 | WT-M/WT-M/WT-M/WT-M/BA.5-M/XBB.1.5-M/XBB.1.5-N/KP.2-M/KP.2-M/LP.8.1-M |
| MICH 19 | 33 | O | White | 7 | 3 | 1 | 1 | 1 | 1 | 784 | 840 | 34 | 90 | WT-M/Wt-M/WT-M/BA.5-U/XBB.1.5-U/KP.2-U/LP.8.1-M |
| MICH 20 | 57 | F | White | 6 | 3 | 1 | 1 | 0 | 1 | 1138 | 1196 | 27 | 85 | WT-P/WT-P/WT-M/BA.5-M/XBB.1.5-P/LP.8.1-M |
