# Supplementary figures and images for: LP.8.1-directed COVID-19 mRNA vaccines durably boost neutralizing antibodies and mitigate ancestral immune imprinting
Source: PLoS Pathog. 2026 May 11;22(5):e1014218. doi: 10.1371/journal.ppat.1014218 (PMC13178986; doi:10.1371/journal.ppat.1014218)

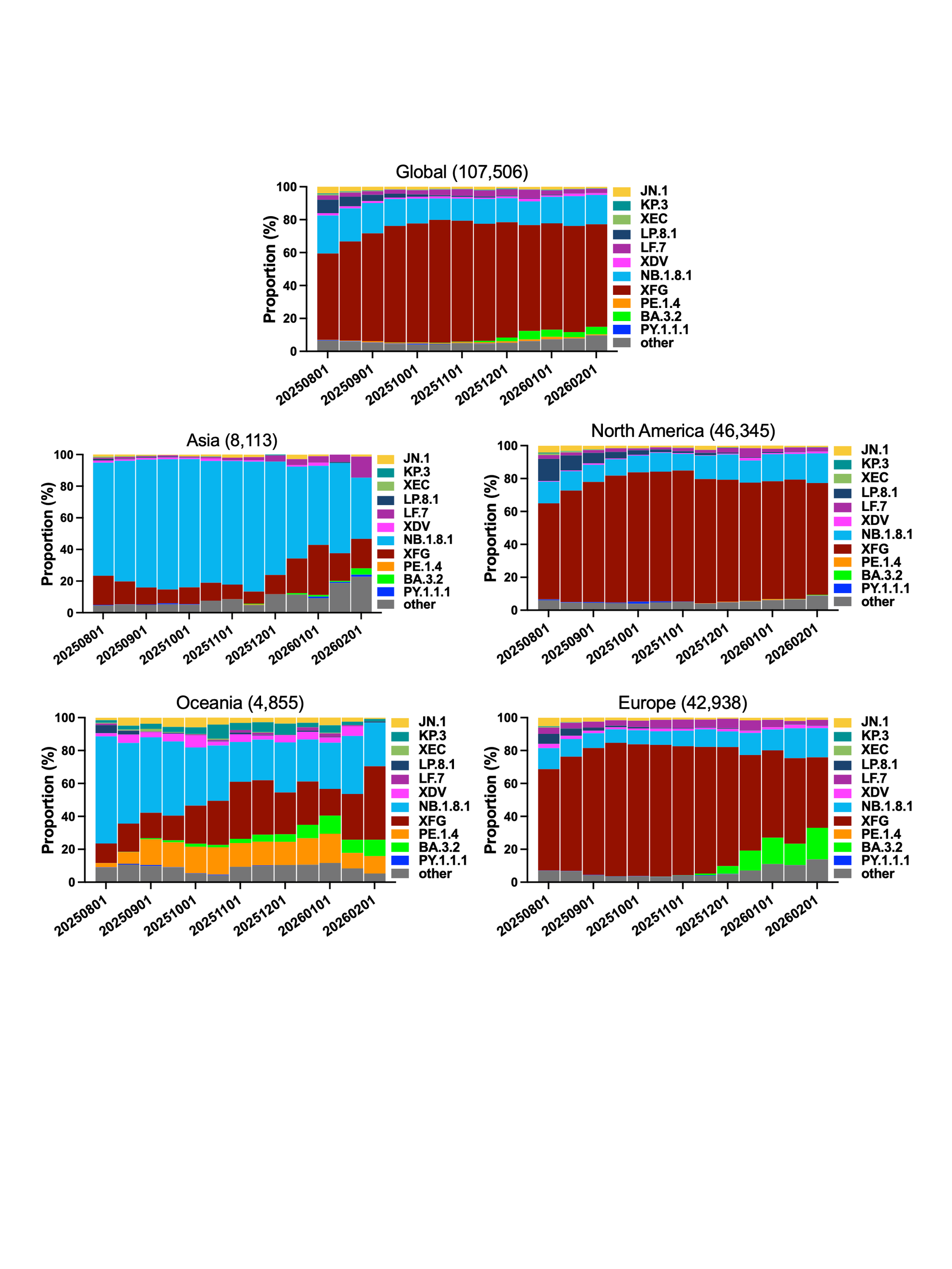

Supplement: S1 Fig — Relative frequencies of SARS-CoV-2 variants from 08/01/2025–02/01/2026 in the indicated regions. Numbers of sequences analyzed in parentheses above each subpanel. Data from GISAID. (TIF) [file ppat.1014218.s005.tif]

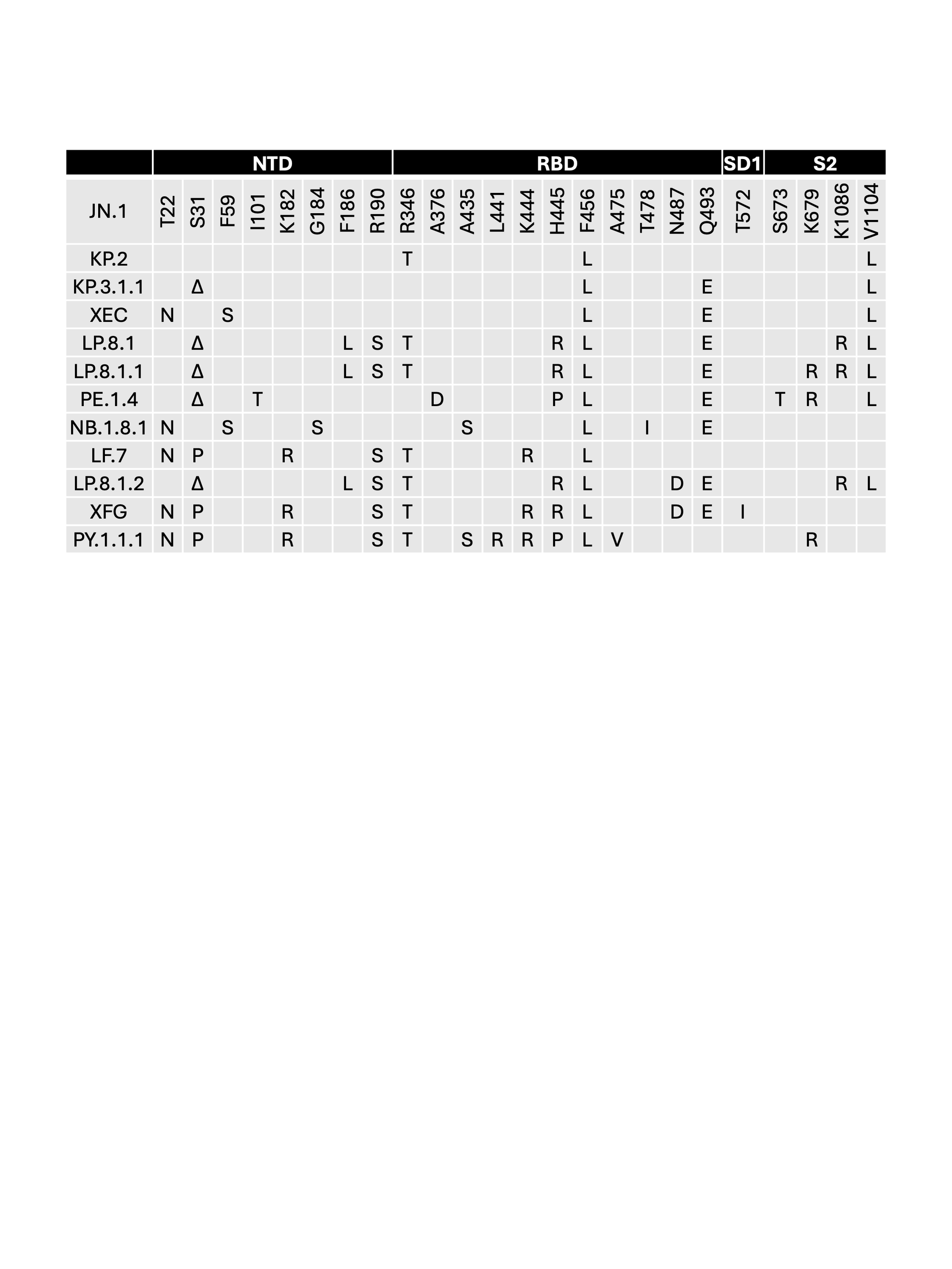

Supplement: S2 Fig — Spike amino acid differences between JN.1 subvariants of interest. BA.3.2 is in the BA.3 lineage, genetically distant from JN.1, and is not included in this table. See Fig. 1A. (TIF) [file ppat.1014218.s006.tif]

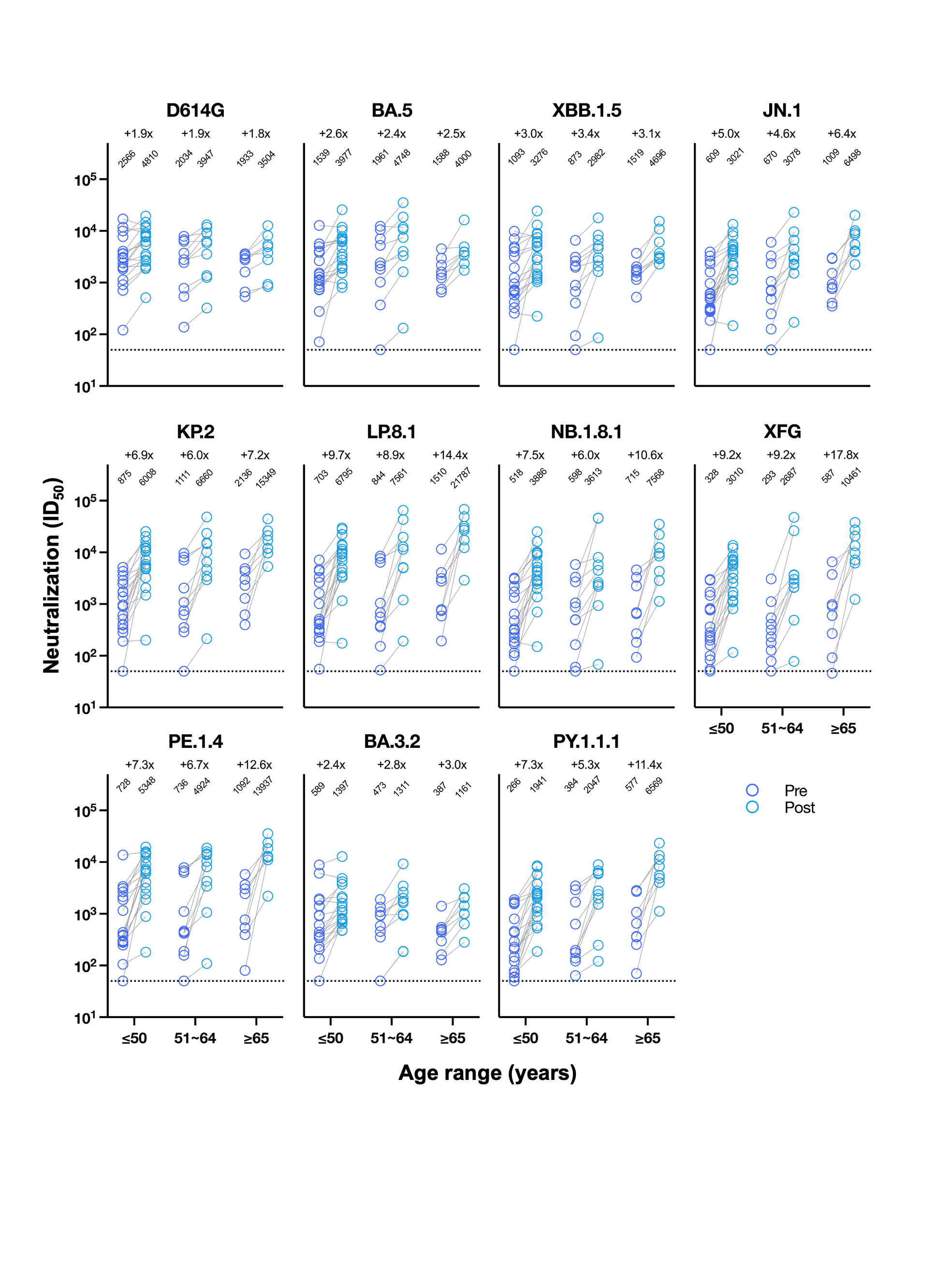

Supplement: S3 Fig — Data are presented as fold changes in neutralization ID50 titers following LP.8.1 MV vaccination for across age groups. Geometric mean titers (GMT) are shown above each sample set, and the fold change from pre- to post-booster is shown above GMTs. MV, monovalent vaccine. n, sample size. The dotted line represents the assay limit of detection (LOD) of 50. (TIF) [file ppat.1014218.s007.tif]
