# Supplementary material for: LP.8.1-directed COVID-19 mRNA vaccines durably boost neutralizing antibodies and mitigate ancestral immune imprinting
Source: PLoS Pathog. 2026 May 11;22(5):e1014218. doi: 10.1371/journal.ppat.1014218 (PMC13178986; doi:10.1371/journal.ppat.1014218)
Supplement: Attachment — Submitted filename: response-to-reviewers.pdf [file ppat.1014218.s010.pdf]

Response to Reviewers

Additional Editor Comments:

Thanks for submitting this valuable study to PLoS Pathogens. One of the reviewers has some constructive minor suggestions. Please incorporate those in a minor revision. I also suggest that you might add a citation and brief comparison/contrast of your results to this recent study in the Discussion (https://www.thelancet.com/action/showPdf?pii=S1473-3099%2826%2900050-2).

We thank the editor for their assessment. We have incorporated the reviewer's comments into the revised manuscript and provided a point-by-point response below. We have also added further discussion of Lasrado et al., 2026 with a comparison of that paper's results to our results in the Results and Discussion section (lines 185-191).

Part I - Summary
Please use this section to discuss strengths/weaknesses of study, novelty/significance, general execution and scholarship.

Reviewer #1: Mellis et al study presents data showing changes in neutralizing titers against different SARS-CoV-2 variants post LP.8.1 booster. They find that there is a broad increase in neutralizing titers post LP.8.1 booster across different SARS-CoV-2 variants, but notably the neutralizing titers are greatest against LP.8.1 and related SARS-CoV-2 variants. This is notable because in prior studies using earlier booster strains neutralizing titers post boost were always highest against D614G variant, which suggest that LP.8.1 booster vaccine might start overcoming immune imprinting caused by the early SARS-CoV-2 variant exposure.

Reviewer #2: The manuscript by Mellis et al is an excellent and important short report about the outcomes of LP.8.1 booster vaccines. A key point is that the booster is finally providing mitigating immune imprinting and providing broad protection. I have several cosmetic corrections and suggestions.

We thank the reviewers for their assessments.

Part II – Major Issues: Key Experiments Required for Acceptance

Reviewer #1: I have no major issues, presented data justifies author conclusions.

Reviewer #2: In the abstract the authors say that this is the first time since 2022 that boost titers were higher against the homologous target than the ancestor, but throughout the paper they say this is the first time that this has ever happened. Which is it?

We appreciate the reviewer pointing out a place where we can clarify the history of COVID-19 vaccines. 2022 was the first year that any non-ancestral strain was included in vaccines authorized in the United States, so we used "since 2022" and "has ever happened" interchangeably. We have clarified this point in the abstract by removing "since 2022" when discussing titer results. We have left in mentions of the history of vaccine formulation updates, which started in the U.S. in 2022.

Fig 1A. The figure twice incorrectly says N478D instead of N487D. The figure neglects the addition of F456L in the XDV lineage. The figure includes LP.8.1.9 for no apparent reason. It was a minor lineage and not included in the experiments, I would remove it if there is no reason for it.

We thank the reviewer for pointing out this typo and we apologize for it. We have updated Fig. 1A to correctly include N487D and F456L as indicated. We note that this typo was only a transcription error during Fig. 1A construction; all plasmids were verified to contain all of the correct mutations by whole-plasmid sequencing prior to pseudovirus generation. For example, the plasmid containing NB.1.8.1 spike contains the F456L mutation, and the plasmid encoding XFG spike contains N487D and does not contain 478D. We have also removed LP.8.1.9 as advised.

For recombinants XDV and XEC the Spike was derived from the parent shown. However, XFG got half of its RBD from LF.7 and the other half from LP.8.1.2. This is why the booster worked so well against XFG (which might be worth mentioning). The way the figure is laid out is inappropriate because it makes it look like 445R, 487D and 493E were acquired through mutation rather than recombination. I would recommend rearranging the figure to show LF.7 and LP.8.1.1 contributing to XFG, perhaps indicating that 445-493 was derived from LP.8.1.1.

We appreciate these important comments. We have updated Fig. 1A to more accurately reflect the phylogenetic history of XFG. Additionally, we have added a new Fig. S2 to show the spike amino acid differences between these closely related JN.1 sublineages.

Part III – Minor Issues: Editorial and Data Presentation Modifications

Reviewer #1: (No Response)

Reviewer #2: Given the high degree of convergent evolution in these lineages, I would recommend making a supplemental figure indicating what residue each lineage has at the heterogeneous positions, at least for the RBD. The data makes a lot more sense when you see it this way rather than simply based on ancestry.

We agree with the reviewer and have added a new supplementary figure accordingly (Fig. S2).

I think it is worth stating explicitly that the ancestral lineage had the highest titer pre-boost, but LP.8 was the highest post-boost. It also think it's probably also worth stating explicitly that that BA.3.2 had by far the lowest titer post-boost.

We have updated the text to clarify this point accordingly.

Journal Requirements:
1) We ask that a manuscript source file is provided at Revision. Please upload your manuscript file as a .doc, .docx, .rtf or .tex.

We have provided the manuscript as a .docx file.

2) Please upload all main figures as separate Figure files in .tif or .eps format. For more information about how to convert and format your figure files please see our guidelines: https://journals.plos.org/plospathogens/s/figures

We have uploaded separate main figure TIF files as adjusted by and downloaded from the PLOS NAAS tool.

3) We have noticed that you have uploaded Supporting Information files, but you have not included a list of legends. Please add a full list of legends for your Supporting Information files after the references list.

We have added a list of Supporting Information figure and table legends to the main manuscript file after the reference list.

4) Please ensure that the funders and grant numbers match between the Financial Disclosure field and the Funding Information tab in your submission form. Note that the funders and grants must be provided in the same order in both places as well.

We have ensured that grant numbers match.
